# Apical and Lateral Canal Sealer Extrusion in Postoperative Periapical Radiographs Shared on Instagram: A Cross-Sectional Image Analysis

**DOI:** 10.7759/cureus.110362

**Published:** 2026-06-06

**Authors:** Hazal Faiz Arslanparcasi, Mehmet Ali Arslanparcasi

**Affiliations:** 1 Endodontics, Harran University, Sanliurfa, TUR; 2 Dental Prosthesis Technology, Faculty of Vocational School of Health Services, Harran University, Sanliurfa, TUR

**Keywords:** apical sealer extrusion, endodontics, instagram, lateral canal sealer extrusion, periapical radiography, root canal treatment

## Abstract

Aim: The aim of this cross-sectional image analysis study was to determine the prevalence of apical sealer extrusion and lateral canal sealer extrusion in postoperative periapical radiographs publicly shared on Instagram and to evaluate the relationship between these two radiographic findings.

Methods: Postoperative periapical radiographs publicly shared on Instagram between January 1, 2025, and December 31, 2025, were evaluated in this study. Following a standardized search using the hashtags #endodontics, #rootcanal, #rootcanaltreatment, and #endodontist, 102 radiographs that met the inclusion criteria were included. Each image was independently assessed by two observers for the presence of apical sealer extrusion and lateral canal sealer extrusion. Interobserver agreement was determined using Cohen's kappa analysis, and the association between apical sealer extrusion and lateral canal sealer extrusion was analyzed using the chi-square test of independence. The level of statistical significance was set at (p < 0.05).

Results: Interobserver agreement was (κ = 0.920) for apical sealer extrusion and (κ = 0.835) for lateral canal sealer extrusion, indicating excellent agreement for both variables. Apical sealer extrusion was detected in 56 cases (54.9%), whereas lateral canal sealer extrusion was identified in 17 cases (16.7%). Apical sealer extrusion was observed more frequently than lateral canal sealer extrusion. However, no statistically significant association was found between apical sealer extrusion and lateral canal sealer extrusion (χ² = 0.507, p = 0.477).

Conclusions: In postoperative periapical radiographs shared on Instagram, apical sealer extrusion was observed at a higher prevalence than lateral canal sealer extrusion. However, no significant association was identified between these two radiographic findings. The findings should be interpreted only within the context of selected postoperative radiographs obtained from social media. Further studies incorporating clinical patient records, standardized imaging protocols, sealer type, obturation technique, and long-term follow-up data may contribute to a more comprehensive understanding of the clinical significance of apical and lateral canal sealer extrusion.

## Introduction

One of the fundamental objectives of root canal treatment is to remove infected pulp tissue and, following biomechanical preparation of the root canal system, to prevent reinfection by sealing the canal space effectively. In this process, three-dimensional obturation of the canals and the achievement of an effective seal are critically important for treatment success [1]. The complex anatomy of the root canal system includes structures outside the main canal, such as lateral and accessory canals, apical ramifications, and canal irregularities. Because these anatomical areas may serve as potential reservoirs for microorganisms and tissue remnants, the use of sealer materials with favorable flowability and sealing properties plays an important role not only in the obturation of the main canal but also in the filling of ramifications and lateral/accessory canals [2-4]. However, the extrusion of sealer materials beyond the apical foramen or their extension beyond the root boundaries through lateral/accessory canals during obturation represents radiographic findings that should be carefully evaluated in terms of periapical tissue responses and treatment prognosis. Although successful root canal treatment requires a homogeneous and dimensionally stable canal filling extending to the apical foramen, the radiographic presence of extruded sealer is considered one of the important indicators of endodontic treatment quality and may be associated with the development of apical periodontitis [5].

However, the effect of sealer extrusion on the prognosis of endodontic treatment remains unclear in the literature. Nevertheless, variables such as sealer type, amount of extrusion, presence of a preoperative periapical lesion, obturation technique, and follow-up duration are important confounding factors in interpreting this relationship. Therefore, sealer extrusion should be regarded not merely as a technical radiographic finding but also as a multidimensional parameter that requires careful biological and prognostic evaluation [6,7].

Periapical radiographs provide important diagnostic information during different stages of endodontic treatment, including the evaluation of canal morphology, verification of working length and preparation, assessment of obturation quality, and monitoring of post-treatment radiographic findings [8]. The apical extent, homogeneity, and extrusion of the root canal filling beyond the root canal boundaries are among the key parameters in the radiographic assessment of treatment quality [9].

The long-term success of root canal treatment is associated with the healing of the periapical tissues and the functional retention of the tooth in the oral cavity. Clinical studies have reported success rates ranging from 74.7% to 85.2% [10]. Traditionally, such radiographic evaluations have been conducted using clinical records and patient follow-ups; however, in recent years, social media platforms have also become widely used for sharing clinical cases, treatment outcomes, and radiographic images in dentistry and endodontics. In particular, postoperative periapical radiographs shared on visually oriented platforms, such as Instagram, may provide a new and accessible data source for cross-sectional evaluation of radiographic findings, including apical and lateral canal sealer extrusion [11,12]. This indicates that digital imaging and online sharing technologies may enable new methodological approaches for the observational assessment of endodontic treatment outcomes.

Unlike traditional clinical databases, social media platforms have become dynamic digital environments in which visual materials from real clinical practice are shared with broad audiences. Dentists and clinical users may share content on Instagram, including clinical photographs, radiographs, and treatment stages. This allows publicly available social media posts to be considered as secondary data sources that may be used for the observational evaluation of specific clinical or radiographic characteristics. However, social media-derived data have ethical and methodological limitations, including selection bias, differences in image quality, limited clinical information, patient privacy, and consent procedures. Therefore, the findings of social media-based studies should not be interpreted as generalizable clinical outcomes, but rather as cross-sectional analyses of selected radiographic content shared in the digital environment [13,14].

Despite these limitations, studies evaluating apical sealer extrusion and lateral canal sealer extrusion together using postoperative periapical radiographs publicly shared on Instagram remain limited in the literature. Although social media-derived radiographs cannot replace clinical datasets, they may provide an accessible source for identifying how specific postoperative radiographic findings are visually represented in publicly shared endodontic cases. In particular, the simultaneous evaluation of apical sealer extrusion and lateral canal sealer extrusion in such images remains limited in the literature. Therefore, a descriptive image-based analysis of these findings may help document their relative radiographic prevalence and clarify whether they tend to appear together in selected publicly available postoperative radiographs. This study aimed to determine the prevalence of apical sealer extrusion and lateral canal sealer extrusion in postoperative periapical radiographs publicly shared on Instagram and to evaluate the relationship between these two radiographic findings. Because information on sealer type, obturation technique, operator-related factors, and clinical follow-up was not available from social media-derived images, the study was designed as a descriptive radiographic image analysis rather than an assessment of treatment quality, prognosis, or material-specific clinical performance.

## Materials and methods

Study design

This study was designed as a social media-based, cross-sectional observational image analysis. Postoperative periapical radiographs publicly shared on Instagram were evaluated using predefined radiographic criteria, and each image was assessed dichotomously for the presence or absence of apical sealer extrusion and lateral canal sealer extrusion.

The study was based solely on anonymous digital radiographic images shared on social media and did not include any clinical patient data. Each image was assessed dichotomously as “absent/present” in terms of the presence of apical sealer extrusion and lateral canal sealer extrusion. During the research process, no personally identifiable data, such as patient name, age, sex, username, profile link, location information, or clinical record information, were recorded.

Data source

All data were obtained from postoperative periapical radiographs shared by publicly accessible accounts on the Instagram platform. The materials used in the study consisted of digital images that had been openly shared on social media by third parties. The researchers did not access any clinical record system and did not collect or process any personal data, such as patient identity, name, age, file number, or similar information. Therefore, all analyses were performed using anonymized visual data.

Data collection process

Content shared on the Instagram platform between January 1, 2025, and December 31, 2025, was systematically screened. The data collection and analysis process was conducted on January 15, 2026.

Sample size

The sample size was calculated for the chi-square test of independence using G*Power software (v3.1) (Heinrich Heine University Düsseldorf, Düsseldorf, Germany). Based on a medium effect size (w = 0.30), 80% statistical power (1 − β = 0.80), and a 5% significance level (α = 0.05), the minimum required sample size was determined to be 88 cases. To increase the reliability of the analysis and to compensate for potential case losses due to exclusion criteria, a total of 102 cases were included in the study.

Sampling method

To minimize the effect of platform algorithm-based personalization, all searches were performed using the same device, in the browser’s incognito mode, and with a standardized search strategy. The data were obtained using the hashtags #endodontics, #rootcanal, #rootcanaltreatment, and #endodontist on the Instagram platform. Posts under each hashtag were evaluated by considering the date range from January 1, 2025, to December 31, 2025. Postoperative periapical radiographs that met the inclusion criteria were recorded in a Microsoft Excel (Microsoft Corporation, Redmond, Washington, USA) database and arranged chronologically according to their publication dates. The sample was created using a consecutive selection approach, starting from the most recent posts and proceeding backward. Duplicate posts belonging to the same case or identical images encountered again under different hashtags were considered as a single case. The data collection process was terminated once the predetermined sample size had been reached.

Inclusion and exclusion criteria

The eligibility criteria used for image selection are summarized in Table 1. Postoperative periapical radiographs were included when they showed the final root canal filling, the tooth and apical region were clearly visible, and the image quality was adequate for radiographic assessment. Images were excluded when they did not represent postoperative periapical radiographs, when the evaluated tooth or apical region could not be clearly distinguished, or when duplicate posts belonged to the same case. Because the study was based on publicly shared postoperative radiographic images, sealer type, obturation technique, and activation procedures could not be reliably verified and, therefore, were not used as inclusion or exclusion criteria. Accordingly, the eligibility criteria were restricted to radiographic accessibility, postoperative status, image type, and duplicate control.

**Table 1:** Inclusion and exclusion criteria.

Criterion type	Criterion
Inclusion	Postoperative periapical radiographs publicly shared on Instagram showing the final root canal filling
Inclusion	Images in which the tooth to be evaluated and the apical region were clearly visible
Inclusion	Radiographs with adequate image quality
Inclusion	In radiographs containing more than one endodontically treated tooth, only the tooth that was clearly the focus of the post and whose apical region was assessable was eligible for analysis
Exclusion	Preoperative and intraoperative images
Exclusion	Cone beam computed tomography (CBCT) images
Exclusion	Panoramic radiographs
Exclusion	Clinical photographs or intraoral images only
Exclusion	Low-resolution or non-assessable images
Exclusion	Images in which the endodontically treated tooth to be evaluated or the apical region could not be clearly distinguished
Exclusion	Radiographs containing more than one endodontically treated tooth in which the tooth to be evaluated could not be clearly identified
Exclusion	Duplicate posts belonging to the same case

Radiographic assessment

All images were independently evaluated by two dentists, consisting of a research assistant (H.F.A.) and a lecturer (M.A.A.). Before the assessment, the observers underwent a calibration process by discussing the evaluation criteria on 10 sample radiographic images that were not included in the study sample.

Each case was evaluated dichotomously according to the presence or absence of lateral canal sealer extrusion and apical sealer extrusion. A radiopaque sealer appearance extending beyond the apical foramen into the periapical region was considered apical sealer extrusion. A radiopaque sealer appearance separating laterally from the root canal filling and extending beyond the root boundaries or along a lateral/accessory canal pathway was evaluated as lateral canal sealer extrusion.

Interobserver agreement and data generation

Interobserver agreement was assessed separately for the variables of apical sealer extrusion and lateral canal sealer extrusion using Cohen’s kappa coefficient. Cohen’s kappa values were interpreted as follows: 0.00-0.20, poor agreement; 0.21-0.40, fair agreement; 0.41-0.60, moderate agreement; 0.61-0.80, good agreement; and 0.81-1.00, excellent agreement. After the independent assessments were completed, cases with discrepancies between the two observers were re-evaluated, and a consensus decision was reached through discussion. The final dataset generated based on this consensus was used for the statistical analyses.

Statistical analysis

Statistical analyses were performed using IBM SPSS Statistics software (version 27, IBM Corp., Armonk, NY, USA). Before the analysis, interobserver agreement for the variables of apical sealer extrusion and lateral canal sealer extrusion was evaluated using Cohen’s kappa coefficient.

The prevalence of apical sealer extrusion and lateral canal sealer extrusion was calculated as numbers and percentages using descriptive statistics. The association between apical sealer extrusion and lateral canal sealer extrusion was evaluated using the chi-square test of independence. Whether the expected cell frequencies in the contingency table met the assumptions of the chi-square test was checked. The level of statistical significance was set at p < 0.05 for all tests.

Ethical considerations

This study was based on an image-based analysis of postoperative periapical radiographs publicly shared on the Instagram platform. During the research process, no data containing direct or indirect personal identifiers, such as patient name, age, sex, username, profile link, location information, or clinical record number, were recorded. The images were evaluated only for the presence of apical sealer extrusion and lateral canal sealer extrusion and were not associated with any patient, user, clinical account, or health record. No data were collected directly from human participants, no contact was made with patients or users, and no clinical intervention was performed. Therefore, the study was considered a secondary analysis of visual material processed in a manner that involved publicly available content and did not include personally identifiable data.

## Results

A total of 102 postoperative periapical radiographs were included in the study. Interobserver agreement was assessed using Cohen’s kappa coefficient. The kappa values were κ = 0.920 for apical sealer extrusion and κ = 0.835 for lateral canal sealer extrusion, indicating excellent agreement for both variables (p < 0.001) (Table 2).

**Table 2:** Interobserver agreement (Cohen’s kappa analysis).

Variable	Kappa	SE	Z	p-value	Interpretation
Apical sealer extrusion	0.920	0.039	9.292	<0.001	Excellent
Lateral canal sealer extrusion	0.835	0.072	8.437	<0.001	Excellent

Apical sealer extrusion was detected in 56 cases (54.9%), whereas lateral canal sealer extrusion was identified in 17 cases (16.7%). Apical sealer extrusion was observed at a higher prevalence than lateral canal sealer extrusion (Table 3). The cross-tabulated distribution of apical and lateral canal sealer extrusion findings is presented in Table 4.

**Table 3:** Prevalence of apical and lateral canal sealer extrusion.

Variable	Present, n (%)	Absent, n (%)
Apical sealer extrusion	56 (54.9)	46 (45.1)
Lateral canal sealer extrusion	17 (16.7)	85 (83.3)

**Table 4:** Cross-tabulation of apical sealer extrusion and lateral canal sealer extrusion findings.

	Apical: No	Apical: Yes	Total
Lateral: No	37	48	85
Lateral: Yes	9	8	17
Total	46	56	102

The association between apical sealer extrusion and lateral canal sealer extrusion was analyzed using the chi-square test of independence. The analysis revealed no statistically significant association between the two variables (χ² = 0.507, df = 1, p = 0.477; Table 5). The effect size was very small (φ = 0.071).

**Table 5:** Chi-square test for the association between apical sealer extrusion and lateral canal sealer extrusion.

Test	Value	df	p-value
Pearson Chi-square	0.507	1	0.477

## Discussion

In this cross-sectional image analysis study, 102 postoperative periapical radiographs publicly shared on Instagram were evaluated; apical sealer extrusion was detected in 56 cases (54.9%), whereas lateral canal sealer extrusion was identified in 17 cases (16.7%). Apical sealer extrusion was observed at a higher prevalence than lateral canal sealer extrusion; however, no statistically significant association was found between the two radiographic findings (χ² = 0.507, p = 0.477). The excellent level of interobserver agreement, with κ = 0.920 for apical extrusion and κ = 0.835 for lateral extrusion, supports the consistency of the criteria used during the evaluation process. Nevertheless, it should be considered that these findings should be interpreted only within the context of selected postoperative radiographs shared on a social media platform and do not directly represent the general clinical population. Importantly, these findings should not be interpreted as a direct comparison of sealer materials or obturation techniques, because such information was not available from the analyzed social media posts.

Apical sealer extrusion has been reported in the literature as a radiographically detectable finding after root canal obturation, with a prevalence ranging from 6.9% to 58% [5]. This condition is associated with various technical and anatomical factors, such as inadequate apical control, errors in working length, pressure applied during obturation, flowability of the sealer material, and the width of the apical foramen. Although it has been stated that extruded sealer material may affect the periapical tissue response and play a role in treatment prognosis, some studies, particularly those involving bioceramic sealers, have reported high success rates despite the presence of extrusion. For example, in retrospective analyses of cases treated with iRoot SP, the healing rate in cases with extrusion was reported to be 95.8%, and partial resorption of the extruded material was observed during follow-up [15]. Similarly, in studies using bioceramic sealers such as TotalFill, more favorable apical healing findings, increased radiopacity during follow-up, and low resorption rates have been reported compared with AH Plus [16]. However, the clinical effect of extrusion may vary depending not only on the presence of extruded material but also on factors such as the type, amount, and localization of the extruded material and the initial periapical status. Since the present study did not include data on sealer type, obturation technique, working length, operator experience, or clinical follow-up, it is not possible to draw a direct inference regarding the effect of apical sealer extrusion on clinical prognosis [17]. This limitation is particularly important because current clinical data indicate that the prognostic effect of sealer extrusion may vary not only according to the presence of extrusion but also according to the initial periapical status and the evaluation criteria. In their retrospective cohort study evaluating endodontically treated teeth with unintended sealer extrusion, Bamrungwong et al. reported that success rates varied markedly depending on the success criteria used and that, according to strict criteria, the main factor affecting treatment success was the size of the periapical lesion. Therefore, the fact that the present study evaluated only the presence of postoperative radiographic extrusion limits prognostic interpretations [18].

Lateral canal sealer extrusion may be considered a radiographic finding associated with the complex anatomy of the root canal system. The concentration of lateral and accessory canals, particularly in the apical third, may allow sealer material to spread laterally through these anatomical pathways [4]. The obturation technique and the physical properties of the sealer material are also among the important factors that may affect lateral canal sealer extrusion. This interpretation is supported by a recent in vitro study showing that the obturation technique significantly affected the penetration of AH Plus Bioceramic sealer into lateral canals in 3D-printed lateral incisor models. In that study, the warm vertical condensation technique provided greater penetration into lateral canals compared with single-cone and cold lateral condensation techniques. Therefore, the rate of lateral canal sealer extrusion detected in the present study may be associated not only with anatomical variations but also with the obturation technique used and the flow properties of the sealer material [19]. Indeed, it has been shown that the vertical condensation technique may provide a higher filling rate in accessory canals compared with lateral condensation, and in some studies, the rate of complete gutta-percha filling has been reported as 50.33% [20]. Although the radiographic appearance of a “lateral puff” may be interpreted in some approaches as effective sealer penetration or filling of complex canal anatomy, it is not appropriate to consider this finding alone as an indicator of optimal obturation or clinical success. Histopathological data indicate that vital tissue remnants may be present in lateral canal areas and that these regions may be associated with an inflammatory response; therefore, the radiographic appearance of lateral extrusion does not always guarantee biological healing or effective sealing [21]. For this reason, lateral canal sealer extrusion may be regarded as a radiographic reflection of complex canal morphology; however, it should not be accepted as a standalone determinant of clinical success [2]. Although the lateral canal sealer extrusion rate of 16.7% detected in the present study is noteworthy in this respect, the clinical significance of this finding should be interpreted cautiously because variables such as sealer type and obturation technique were unknown.

In this study, no statistically significant association was found between apical sealer extrusion and lateral canal sealer extrusion (p = 0.477). This result suggests that although the two findings represent similar appearances of material extrusion, they may arise through different anatomical and technical mechanisms. While apical sealer extrusion may be more closely associated with apical foramen morphology, working length, apical patency, obturation pressure, and apical control, lateral canal sealer extrusion may be related to accessory canal patency, lateral canal localization, sealer viscosity, and the dynamics of lateral spread [22]. The three-dimensional and variable anatomical structure of the root canal system may contribute to the emergence of these two types of extrusion as independent radiographic findings. However, due to the cross-sectional and image-based design of the study, it is not possible to establish a causal mechanism between these findings.

Social media platforms such as Instagram provide an accessible data source for the observational evaluation of endodontic findings by enabling postoperative periapical radiographs to be shared with broad audiences. However, clinical cases shared on social media are generally not randomly selected cases. Since cases considered successful, striking, aesthetic, or educationally valuable are more likely to be shared, such data may include substantial selection bias and may not represent the general clinical population [12]. Although the increasing use of social media in endodontic education enhances the visibility of clinical examples, the level of scientific evidence, accuracy, and standardization of information presented on these platforms may vary. Therefore, radiographic findings derived from social media should be interpreted cautiously when making clinical inferences and should not be regarded as direct evidence that can replace traditional clinical research data [23].

This study has several limitations. First, the study was based not on clinical patient records but on postoperative periapical radiographs publicly shared on Instagram. Therefore, variables such as sealer type, including whether the material was bioceramic- or zinc oxide eugenol-based, obturation technique, use of activation procedures, instrumentation system, working length, operator experience, tooth type, root canal anatomy, demographic characteristics, and clinical follow-up outcomes could not be evaluated. This limitation prevents material-specific or technique-specific interpretation of the findings and restricts the conclusions to the radiographic presence or absence of extrusion in the selected images. The absence of demographic characterization and control is an inherent limitation of social media-derived datasets and may further affect the representativeness and generalizability of the findings. It has been reported that awareness of bioceramic sealers has increased in current clinical practice and that the clinical use of these materials has become widespread; therefore, the unknown type of sealer used should be considered an important factor limiting the interpretation of apical and lateral sealer extrusion findings in the present study [24]. Second, the analysis was performed only on two-dimensional periapical radiographs. Since periapical radiographs represent three-dimensional dental and periapical anatomy in two dimensions, they may involve limitations related to the superimposition of anatomical structures, geometric distortion, and imaging angulation. Therefore, the three-dimensional location, volume, and actual extent of apical or lateral sealer extrusion could not be precisely determined in the present study [25]. Third, the angulation, resolution, contrast, and overall image quality of the images shared on social media were not standardized. In addition, the Instagram algorithm and users’ posting preferences may have introduced selection bias in the images included in the study. Nevertheless, the use of predefined evaluation criteria, assessment by two independent observers, excellent interobserver agreement, and an original design evaluating apical and lateral canal sealer extrusion together in social media-derived postoperative radiographs may be considered among the strengths of this study.

## Conclusions

In this cross-sectional image analysis study, apical sealer extrusion was found to be more frequently observed than lateral canal sealer extrusion in postoperative periapical radiographs publicly shared on Instagram. However, no statistically significant association was detected between apical sealer extrusion and lateral canal sealer extrusion. The findings should be interpreted within the context of selected radiographic images derived from social media, and no direct inference regarding clinical prognosis should be made. Future studies incorporating clinical patient records, standardized radiographic protocols, sealer type, obturation technique, and long-term follow-up data may contribute to a more reliable evaluation of the clinical significance of these radiographic findings.
